# Clinical Experience with Pituitrin in Obstetrics

**Published:** 1914-05

**Authors:** L. F. Anderson

**Affiliations:** Obstetrician, St. Marys Maternity Hospital, Buffalo, N. Y.


### CLINICAL EXPERIENCE WITH PITUITRIN IN OBSTETRICS.

BY L. F. ANDERSON, M. D., OBSTETRICIAN
St. Marys Maternity Hospital,
Buffalo, N. Y.

---

THE value of the extract of the posterior or infundibular portion of the pituitary gland in uterine inertia has been under the consideration of the medical profession approximately for the past four years. That it is of very great value has been fully established as evident from the immense volume of literature covering the subject.

The pituitary gland or pituitary body has always been a puzzle to the anatomist and physiologist. Composed, as it is, of two lobes, it is believed that the earliest researches into the function of the gland failed to develop any substantial discovery owing to the fact that extracts of the whole gland were employed. In all likelihood Dale was the first person to discover that pituitary extract, injected intravenously into a rabbit, would cause contraction of the uterus. This discovery occurred in 1906, and in 1909 Bell read the first clinical paper on the value of infundibular extract in shock, uterine atony, and intestinal paresis. He reported a rise in blood pressure following the injection and powerful contractions in the puerperal, pregnant, or menstruating uterus. It was particularly successful in post-partum hemorrhage from atonic uteri, and was very valuable in Cesarean section.

Frankl-Hochwart and Frohlich of the Pharmacological Institute of The University of Vienna, did considerable work also in directing attention to the uterine action of the extract. Later Foges and Hofstatter in Germany obtained very favorable results from the use of Pituitrin (pituitary extract) in post-partum hemorrhage. Afterwards Hofbauer, made the first attempt to use the drug in the promotion of labor pains, and numerous reports of the value of Pituitrin in labor soon followed.

Observations of Foges and Hofstatter are especially interesting. They administered Pituitrin in sixty-three cases of atony, intravenous or intramuscular injections of 1 to 2 Cc., diluted with 20 Cc. of normal horse serum being given. A few minutes later a very light massage of the previously inert uterus would stimulate the organ to sudden contraction, in which state it remained for a long time, thus preventing hemorrhage. They say in conclusion, "it is certain that in these cases Pituitrin is not only equal but superior to ergotin, as regards both the intensity and the duration of the contraction." **(Zentralb fuer Gynaekologie, 1910, No. 46).**

Dr. Emil Vogt of the Royal Gynecological Clinic at Dresden, gives his experience in the **Munchener Medizinische Wochenschrift,** Dec. 19 1911, as follows:

"The oxytocic action of Pituitrin (P. D. & Co.) at this clinic was observed in over one hundred cases. After the rupture of the fetal membranes, in the second stage of labor, the physiologic effect of Pituitrin is the most pronounced; the contractions of the uterus follow each other much more rapidly and energetically, and the intervals between pains are decreased. Individually the pains are not more severe, so far as suffering is concerned, even in the case of sensitive women, than they would be in a normal delivery.

"In half of the cases the Pituitrin was administered in the second stage of labor. It failed only once; in all other instances its action was very pronounced. And although we encountered a great many cases of narrow pelvis in Dresden, from 40 to 50 per cent., it was not necessary to have recourse to forceps delivery in a single instance in which Pituitrin was employed. Nor did we observe any tetanus uteri and the consequent injury to the child.

"In a number of miscarriages in which we also put the agent to a practical test, it proved rather unsatisfactory. In three out of seven cases there was a noticeable effect; but twice, in cases of miscarriage in the third and fifth month, it failed utterly, despite repeated injections.

"We used Pituitrin seven times in placenta praevia lateralis, with good success, after the rupture of the membranes. The pains were intensified and the labor shortened, which was in accordance with the principal indication to empty the uterus as soon as possible.

"Our experiences coincide with the clinical and experimental observations of E. Kehrer and show that there is no reason why Pituitrin should not be generally employed in obstetric practice.

"In the second stage of labor the action of Pituitrin is prompt and certain. It serves to accelerate normal deliveries, and may be used to combat secondary insufficiency of labor pains even in cases of narrow pelvis. It is most active in the second stage of labor, but may also be used to advantage in the stage of dilatation.

"According to our experience, Pituitrin is the most ideal oxytocic we possess to-day."

Experiences of other investigators practically coincide with this report.

Among others, Dr. Alfred Studeny of the Moravian lying-in institution at Bruenn may be quoted from the **Wiener Klinische Wochenschrift, Dec. 21. 1911**, as follows:

"In summing up our experience we would designate Pituitrin as the most reliable oxytocic agent known at the present time. Its action is most pronounced in the last stage of labor. In the placental period it seems to prevent uterine atony, but in an existing atony the ergot preparations are still preferable. It is non-toxic and does not require care in regard to dosage. As an oxytocic it represents an exceedingly valuable addition to the obstetric materia medica."

This observer treated eighty-one cases with Pituitrin, which, with the exclusion of instrumental deliveries, amounted to forty cases of spontaneous labor. Thirty-five were occipital presentations, one face, and four pelvic presentations. Twenty-three were primiparae. In the first stage of labor Pituitrin was administered six times with a pronounced oxytocic action. In the remainder the preparation was administered in the last stage of labor. These were mostly protracted deliveries with an average duration of forty-two to thirty-four hours in primiparae and multiparae respectively. The results were excellent, and rarely required a repetition of the dose.

It is now generally conceded that Pituitrin may be safely administered to the parturient woman. It does not produce deleterious effects apparently upon mother or child; in fact it seems to improve the circulation of both, and stimulates the renal function of the new-born child. The mother appears to be in better condition following the administration of Pituitrin and the child is more easily resuscitated if we may judge from the reports of European investigators.

The usual quantity of Pituitrin which is required to exert its full therapeutic effect varies from 1-2 to 2 Cc. although it is most satisfactory to administer approximately 1 Cc., and repeat if necessary. The preparation should be administered hypodermatically, as there is practically no evidence to show that it has any therapeutic value when administered internally.

Pituitrin differs markedly from ergot preparations in regard to the type of uterine contraction produced. In the former, with the proper dosage, the contractions are regular and rhythmical in character, and the uterus is not set in tetanic contraction. Results usually follow the injection of Pituitrin in from three to five minutes.

The indications for the administration of Pituitrin are identical with those requiring a hastening of labor. Thus, it is of value in uterine inertia resulting in exhaustion of the mother and danger to the child; lateral placenta praevia after rupture of the membranes; cardiac disease and threatened eclampsia on the part of the mother, slightly contracted pelvis, and hemorrhage of all kinds.

Pituitrin should not be employed when Cesarean section is indicated on account of pelvic deformity. It is a dangerous preparation to use in cases in which powerful uterine contraction would be a menace to mother or child.

Dr. Oscar Bondy of the Gynecological Clinic of the University of Breslau, reports having employed Pituitrin in the production of labor pains in ten cases, with successful results in eight, partial success in one, and negative results in one. The last case was that of an elderly primipara with a breech presentation, a very large child, and insufficient labor pains After labor had continued for fifty-four hours, the pains practically ceased, and 1 Cc. of Pituitrin was administered. Slight pains resulted, but, as these soon ceased, labor was terminated by surgical means. The second case was an elderly primipara, thirty-four hours in labor, with only slight pains. Powerful contraction followed the administration of Pituitrin and birth occurred without assistance.

In the eight successful cases observed by Bondy, the duration of labor before Pituitrin was employed varied between twenty-three hours and forty-eight hours, with an average of thirty-six hours. After injections of Pituitrin, labor was terminated in from five to sixty minutes with an average of twenty-eight minutes. The author in the "Berliner Klinische Wochenschrift, August 7th, 1811, reports these cases in full and concludes as follows:

"The above includes cases of primiparae and multiparae, all cranial presentations, and one case of twins. It is evident that the labor period in all cases had been prolonged considerably. The fetal membranes in most cases were ruptured; the os uteri was considerably but not completely dilated. Parturition quickly took place after the administration of Pituitrin. If we consider the first figures as representing approxi mately the duration of the first stage of labor (dilatation period), and the others the duration of the second stage of labor (expulsion period), we see that the average of thirty-six hours in labor before Pituitrin was injected is two to threefold the normal period of the first stage of labor, and that the average of twenty-eight minutes of the second stage after Pituitrin is one-fourth to one-half that of the normal second stage of labor.

"We have had no unpleasant experiences and never observed an injury to the child. As regards trouble in the third stage of labor, we have observed only now and then a slight loss of blood after the expulsion of the placenta, which follows spontaneously. This can be avoided by the administration of an ergot preparation after the placenta is ejected. The dose of Pituitrin is 1 Cc. subcutaneously, repeated as indicated. For general practice the ampoules containing 1 Cc. are recommended.

Dr. Hans Hermann Schmid, first assistant of the Obstetrical Clinic of the University of Prague, in the "Gynaecologische Rundschau, No. 15, 1911," gives his experience with Pituitrin as follows:

"I administered Pituitrin, of Parke, Davis & Co.'s manufacture, thirteen times in cases of post-partum hemorrhage, five times in Caesarean section, and fifteen times as an oxytocic. After Pituitrin was introduced into the clinic the use of ergot preparations was abandoned, and their absence was not felt. I agree with Blair and Bell that the extract of the pituitary gland will henceforth be indispensable in the obstetrical outfit; those who are aware of the unreliability of ergot preparations must be highly delighted to know that they possess in Pituitrin a preparation that will permanently displace ergot in obstetrics.

"After ascertaining that Pituitrin may be safely injected immediately after the birth of the child, and that, unlike the ergot preparations, it is not liable to produce any disturbances of the placental period, I went a step further and employed it as agent to bring on labor. For this purpose Pituitrin was invariably injected subcutaneously in the Prague clinic, being administered alone in fifteen cases and in connection with an opiate in eight. Of these twenty-three cases there were nine of weak fetal heart sounds, in all of which the umbilical cord was wound about the neck from one to three times. One luetic child was born dead, and one had to be delivered by means of forceps, but the rest were quickly resuscitated after spontaneous delivery.

"I believe it is better to have recourse to Pituitrin than to employ a dilator, as the introduction of the latter is always accompanied by danger of infection. I regard Pituitrin not only as a reliable means for combating atonic post-partum hemorrhage, but as an oxytocic that is superior to all others. Incidentally I have never seen any untoward after-effects following the use of Pituitrin; on the contrary, patients seem to recover much more rapidly from the hardships of labor after its administration.

"Pituitrin is the best and most reliable of all the preparations in use for the treatment of post-partum hemorrhage; it not only surpasses all ergot preparations as regards activity, but is superior to them in another respect; it can be given, without danger, before the placenta is expelled. Furthermore, it is the only certain and safe oxytocic medicament that can be used to advantage in cases in which the forceps or a dilator has heretofore been employed. The rapid delivery of the placenta and surprisingly small post-partum loss of blood are desirable after-effects. The only undesirable after-effects observed in some of the cases (25 per cent.) were after-pains."

The foregoing quotations indicate the opinion of physicians in the large foreign clinics in regard to Pituitrin in the practice of obstetrics. At the present time the literature covers several thousand cases with a uniform success attending Pituitrin therapy that is surprising indeed. Sufficient has been learned concerning this valuable preparation to enable its successful employment in general practice, as well as in hospital clinics. At the present time Pituitrin forms a very valuable aid to the practitioner and should form a part of the supplies in every obstetrical bag.

A series of ten cases occurring in my practice indicate the favorable action of Pituitrin in cases of difficult confinement. These may be given in detail as follows:

Case 1. A. B., primipara, aged 22, in labor twelve hours. dilatation complete but head high up. At 6.15 p. m., 15 minims of Pituitrin were given hypodermically, a small amount of chloroform administered, and the membranes artificially ruptured. Pains before injection about five minutes apart, not very strong. After injection pains strong, three minutes apart. Manual dilatation of perineum to prevent tear. At 6.40 child was born, weight 7½ lbs. Uterus well contracted, cervical ring firm. No tear, puerperium uneventful. Vertex presentation.

Case 2. Annie R., aged 16. Dilatation complete at 7.15 a. m. Membranes bulging, head low down. 15 minims Pituitrin injected at 7.18 a. m. Child born at 7.35 a. m. Slight manual dilatation of perineum and small amount of chloroform. Puerperium uneventful. In labor seven hours before ecbolic was administered. Vertex presentation.

Case 3. Jennie G., aged 14. In labor nine hours. Pains weak and irregular, about five to eight minutes apart. Dilatation at 11.50 p. m. nearly complete. 20 minims Pituitrin at 11.55 p. m. Pains then became strong, and regular, about two minutes apart; chloroform and manual dilatation of perineum. At 12.20 a. m., May 24th, child born, weight 8¼ lbs. No tear. Recovery uncomplicated. Vertex presentation.

Case 4. Alice J., aged 26, primipara, in labor fourteen hours. At 2.00 a. m. dilatation nearly complete. Pains timed until 2.40 a. m., 10 to 8 minutes seperated, weak, no advance of head. At 2.40 a. m., 20 minims Pituitrin administered, and membranes ruptured. Chloroform in small amounts. At 3.25

a. m., child born, weight 7¾ lbs. First degree tear;one silk worm gut suture necessary. Recovery uncomplicated. Vertex presentation.

Case 5. Minnie R., aged 18, primipara, vertex presentation, in labor twenty-two hours. Cervix rigid, dilatation one-half complete. Patient exhausted, P. 118, R. 28, T. 98.2. Pains weak and very irregular, three to fifteen minutes separated. At 12.40 a. m., 20 minims Pituitrin administered. In three minutes thereafter pains became strong, two to three minutes apart. Manual dilatation of perineum. At 12.58 child was born, weight 8¼ lbs. Puerperium uneventful.

Case 6. Mary A., aged 14, primipara, seven hours in labor. Breech presentation. Had severe nephritis during last three months of pregnancy. At 6.00 p. m., dilatation size of a dollar. Pains strong and four to five minutes separated. No advance. 15 minims Pituitrin at 6.30 p. m. At 7.10 p. m., child born, weight 7¼ lbs., no tear. Head was easily extracted. Puerperium uncomplicated.

Case 7. Mary B., aged 15; breech presentation, in eighth month of pregnancy. Suffering from nephritis for four months, last few days had temperature ranging from 100 degrees F. to 102 F., also ocular disturbances. Pains at 2.00 p. m., strong, five minutes separated. Dilatation size of fifty cent piece. 20 minims of Pituitrin given. At 2.40 p. m., child born, still-birth. Recovery uncomplicated.

Case 8. Agnes S., aged 20, primipara, twenty hours in labor. At 9.30 a. m., dilatation size of ten cent piece. 15 minims Pituitrin with no appreciable effect. At four p. m., dilatation nearly completed, pains weak, four minutes separated. 20 minims Pituitrin at 4.00 p. m. At 4.30 p. m. child born. Vertex presentation.

Case 9. May Z., aged 28, multipara, in labor five hours. Dilatation nearly complete at 5.00 p. m. Had always had long labors (5th pregnancy). 20 minims Pituitrin at 5.05 p. m. At 5.15 p. m., child born, weight 8¾ lbs. Recovery uneventful.

Case 10. Celia R., aged 24, primipara, vertex presentation, in labor fifteen hours. Small pelvis, general nutrition poor. At 2.40 a. m., dilatation complete, pains strong, six to seven minutes separated. 20 minims Pituitrin at 3.00 a. m. Child born at 3.25 a. m. Second degree tear. Two silk worm gut sutures.

In each of the foregoing cases the action was prompt and satisfactory in every way. Following its administration the puerperium seemed to be especially free from complicating conditions and discomfort to the patient. The pains brought about by Pituitrin were strong and regular and caused the mother to suffer no more than would have been the case without the use of the oxytocic agent; in fact, in some instances it seemed to be somewhat less. Tetanic contractions of the uterus were not manifested. My experience in over sixty-five cases is that only two failed to respond to Pituitrin injections.

It is apparent that the administration of this product considerably lessens the field for the application of forceps. In some cases Pituitrin renders the application of forceps less difficult and far less dangerous by bringing the head within easy reach. There are numerous striking examples in the literature where inertia of the uterus threatened death of the fetus, and wherein forceps application would have been most difficult because of lack of engagement. Pituitrin caused the head to engage in each instance and brought it within reach of the forceps.

In the ''American Journal of Obstetrics, for September, 1912, Humpstone alone reports eighteen cases of this nature in his experience.

The consensus of opinion at the present time seems to be that Pituitrin is a remedy par excellence for the promotion of labor pains in the second stage of labor, after the os is fully dilated. Opinions differ somewhat as to its value at an earlier stage, although very little success is believed to accompany its use in the initiation of labor pains for the production of miscarriage before the fourth month.

To prevent hemorrhage during Cesarean section most authors are agreed that pituitary extract is of great value. Gynecological surgeons differ somewhat as to the precise moment at which the injections should be given, but they are fairly uniform in their assertion that it successfully causes contraction of the uterus and the prevention of bleeding.

In conclusion it may be said that Pituitrin is an especially valuable preparation in the practice of obstetrics, on account of its producing contractions resembling the natural uterine contractions. It is also a satisfactory heart-tonic and blood pressure raising principal; and has considerable effect on the bladder and kidneys, rendering catheterization after childbirth unnecessary in most cases. It should be handled cautiously in cases of myocarditis and marked nephritis, especially in the presence of high blood-pressure. Nevertheless it still remains an ideal oxytocic agent and deserves recognition by every obstetrician.

### BIBLIOGRAPHY.

1. Schaefer and Oliver: Journal of Physiology, vol. 18, 1895.
2. Howell: Journal of Experimental Medicine, vol, 3, 1898.

3. Falta and Ivcovic: Wien. Klin. Wochenschr., 1909, No. 51.
4. Houghton and Merrill: Jour. Amer. Med. Asso., Nov. 28, 1908.
5. Beck and O'Malley: American Medicine, October, 1909.
6. Wiggers: American Journal of the Med. Sciences, April, 1911.
7. Schafer: American Journal of Physiology, vol. 27, p. 9.
8. Schaefer and Herring: Proceedings of the Royal Society of Medicine, London, vol. 77, 1906.
9. Falta: Wien. Klin. Woch., No. 27, 1909.
10. Falta and Pal: Wien. Med. Wochenschr., 1909, No. 3, and Centralbl. fur Physiol., 1909.
11. v.Frankl-Hochwart and Froehlich: Wien. Klin. Wochenschr., 1909, No. 27.
12. Mummery: Lancet, 1909, vol. 1.
13. Poelinitz: Gulf States Journal of Medicine and Surgery, October 1910.
14. Klotz: Munch. Med. Wochenschr., 1911, No. 21.
15. Williams: Clinical Journal, May 18, 1910.
16. Bate: Long Island Medical Journal, May, 1909.
17. Bab: Wien. Klin. Wochenschr., No. 27, 1911; and Munch. Med. Woch. No. 23, 1911.
18. Ebeler: Med. Klinik, 1911, No. 20.
19. Johnston: Transactions Royal Academy of Medicine, Ireland, vol. xvii, p. 429.
20. Schiff: Zeitschr. fur Klin. Med., 1897, s. 282.
21. Bate: American Practitioner and News, March, 1908, p. 112.
22. Foges and Hofstaetter: Zentralb. fur Gynaekologie, 1910, No. 46.
23. Stern: Zentralb. fur Gynaek., No. 21, 1911.
24. Hercod: La Presse Med. d'Egyte, Nov. 15, 1911.
25. Foges: Wien. Klin. Woch., No. 27, 1911.
26. Etienne and Parisot: Arch. de Med. Experiment., vol. 20, 1908.
27. Frankl-Hochwart and Frohlich: Arch. Pathol. and Pharmakol., 1910, Bd. 63, p. 347.
28. Dale: Jour. Physiol., May, 1907.
29. Bell, W. B.: Brit. Med. Jour., Dec. 4, 1909, p. 1609.
30. Hofbauer: Munchen, med. Wchnschr., March 19, 1912, lix, No. 12.
31. Studeny, Alfred: Wien. Klin. Wchnschr., Dec. 21, 1911, Jahrgang 24, No. 51, p. 1766.
32. Stern, Robert: Berl. klin. Wchnschr., Aug. 7, 1911, No. 32, p. 1459.
33. Bondy, Oscar: Berl. klin. Wchnschr., Aug. 7, 1911, No. 32.

34. Voight: Deutsch. med Wchnschr., Dec. 7, 1911, Jahrgang 37, No. 49, p. 2286.
35. Hahl, C.: Brit. Med. Jour., Dec. 16, 1911, p. 91.
36. Hamm: Deutsch. Med. Wchnschr., March 7, 1912, xxxviii, No. 10, p. 487.
37. Richter, I.: Wien. klin. Wchnschr., 1912, No. 13, p. 480.
38. Hirsch, E.: Munchen. med. Wchnschr., April 30, -912, Jahrgang 59, No. 18, p. 984.
39. Benthin, W., Deutsch. Med. Wchnschr., April 25, 1912 xxxviii, No. 17, p. 823: Munchen. med. Wchnschr., Jahrgang 59, No. 18, p. 994.
40. Hofstatter, R.: Wien. klin. Wchnschr., 1911, No. 49.
41. v. Fellenberg, R.: Wien. klin. Wchnschr., March 7, 1912, xxv, No. 10, p. 390; Korrespondenzblatt f. Schwiezer Artze, 1911, Jahrgang 41, No. 35.
42. Nagy, T.: Centralbl. f. Gynakol., 1912, No. 10.
43. Gusew: Russk. Wratsch., No. 52.
44. Besserer: Medizinische Klinik, April 16, 1912, Jahrgang 8, No. 16, p. 670.
45. Jaeger: Munchen, med. Wchnschr., No. 6.
46. Parisot, J.: and Spire, A.: Med. Rec., April 7, 1912, p. 671.
47. Fries: Munchen. med. Wchnschr., 1911, lviii, 2438.
48. Vogt: Munchen. med. Wchnschr., 1911, lviii, 2734.
49. Schaefer: Munchen. med. Wchnschr., 1912, lix, 75.
50. Schirmer, Gustav: Med. Fortnightly, March 25, 1912.
51. Hager: Berl. klin. Wchnschr., April 15, 1912, No. 16, p. 762
52. Humpstone: American Journal of Obstetrics, Sept. 1912.
53. Dollert: Atlanta Journal-Record of Medicine, Jan., 1913.

125 Jefferson Street. Buffalo, N. Y.

---

Palpation of the Digestive Tract. Hausmann, *Berliner Klin. Woch.,* No. 32, 1913, emphasizes the value of palpation during expiration or the respiratory pause, never during inspiration (but continuous gentle pressure during several inspirations and expirations, facilitates the descent of the hand or fingers and minimizes reflex muscular spasm, though the actual palpation is better performed during the respiratory pause or expiration.—Ed.) The small intestine cannot be palpated except near the cæcum. (This is not absolutely true. Inflated coils and dense growths or contained bodies can be felt, but not definitely located anatomically.—Ed.). By having the patient raise the right leg, the lower leg being extended, the end of the ileum can be felt crossing the psoas, which is brought out as a thick cord and often mistaken for the appendix. The appendix cannot usually be felt unless it is thickened.

A Simple Gram Technique. Snyder, of Toledo (*Annals of Opthalmology,* November, 1912:

METHYL-VIOLET STAIN.

R Melted carbolic acid crystals ..............12.5 c.cm·
Absolute ethyl alcohol .....................25.0 c.cm
Methyl-violet 6 B (Gruebler) .............. 1.0 gram

Dissolve, keep in a warm place for twenty-four hours, and filter.

LUGOL'S SOLUTION.

R Iodine crystals ........................... 1 part
Potassium iodine .......................... 2 parts
Distilled water ...........................300 parts

A drop of formalin solution 1 in 1,000 in distilled water is placed upon the slide. Some of the secretion is evenly mixed with the drop of formalin solution. A drop of absolute alcohol fixes the slide, being superior to the flame method. Three or four drops of distilled water are placed upon the smear, and to the water is added one drop of the methyl-violet stain. This is left for twenty-five seconds, and the slide is washed. Lugol's solution is now added, and after fifteen seconds the smear is decolorized with absolute alcohol. Wash again and counterstain with a 5 per cent. weak fuchsin for five seconds. Wash again, dry and examine.

---

Sterilization of Milk by Electricity. The bacteriological department of Liverpool University has been conducting experiments to ascertain whether electricity can be satisfactorily utilised for sterilization of milk. The milk enters one end of a tripartite tube of definite size at a known fixed level, and during its passage through the tube is acted on by the electric current. Dr. Beattie, the city bacteriologist, has issued a report stating that this method of sterilization is more economical than, and free of some of the objections urged against the older method. Complete destruction of all coli and similar bacilli, resulted. As these organisms are chiefly responsible for summer diarrhoea among children, the milk sterilized by this process is eminently fit for infantile use. The taste of the milk and its nutritive properties are not altered. The tests so far made on tuberculous milk proved that the tubercle bacillus can with certainty be destroyed. As a result of this report the Liverpool Infant Life Preservation Committee—at whose instance these experiments were made—contemplate installing electrical sterilization plant at their depot for treatment of all milk sold by the corporation —*The Medical Officer,* 12th July, 1913.

